# Strigolactone Alleviates the Adverse Effects of Salt Stress on Seed Germination in Cucumber by Enhancing Antioxidant Capacity

**DOI:** 10.3390/antiox12051043

**Published:** 2023-05-04

**Authors:** Changxia Li, Xuefang Lu, Yunzhi Liu, Junrong Xu, Wenjin Yu

**Affiliations:** College of Agriculture, Guangxi University, Nanning 530004, China

**Keywords:** GR24, TIS108, alleviation, antioxidant system, gene expression

## Abstract

Strigolactones (SLs), as a new phytohormone, regulate various physiological and biochemical processes, and a number of stress responses, in plants. In this study, cucumber ‘Xinchun NO. 4’ is used to study the roles of SLs in seed germination under salt stress. The results show that the seed germination significantly decreases with the increase in the NaCl concentrations (0, 1, 10, 50, and 100 mM), and 50 mM NaCl as a moderate stress is used for further analysis. The different concentrations of SLs synthetic analogs GR24 (1, 5, 10, and 20 μM) significantly promote cucumber seed germination under NaCl stress, with a maximal biological response at 10 μM. An inhibitor of strigolactone (SL) synthesis TIS108 suppresses the positive roles of GR24 in cucumber seed germination under salt stress, suggesting that SL can alleviate the inhibition of seed germination caused by salt stress. To explore the regulatory mechanism of SL-alleviated salt stress, some contents, activities, and genes related to the antioxidant system are measured. The malondialdehyde (MDA), H_2_O_2_, O_2_^−^, and proline contents are increased, and the levels of ascorbic acid (AsA) and glutathione (GSH) are decreased under salt stress conditions, while GR24 treatment reduces MDA, H_2_O_2_, O_2_^−^, and proline contents, and increases AsA and GSH contents during seed germination under salt stress. Meanwhile, GR24 treatment enhances the decrease in the activities of antioxidant enzymes caused by salt stress [superoxide dismutase (SOD), peroxidase (POD), catalase (CAT) and ascorbate peroxidase (APX)], following which antioxidant-related genes *SOD*, *POD*, *CAT*, *APX*, and *GRX2* are up-regulated by GR24 under salt stress. However, TIS108 reversed the positive effects of GR24 on cucumber seed germination under salt stress. Together, the results of this study revealed that GR24 regulates the expression levels of genes related to antioxidants and, therefore, regulates enzymatic activity and non-enzymatic substances and enhances antioxidant capacity, alleviating salt toxicity during seed germination in cucumber.

## 1. Introduction

Salt stress is a serious ecological problem that negatively affects crop growth [1], development [2], and productivity worldwide [3]. Meanwhile, salt stress is usually accompanied by oxidative damage due to the excess production of reactive oxygen species (ROS), causing membrane lipids, and thereby inhibiting plant growth and development including seed germination [4], seedling growth [5], plant life, and so on [6]. Additionally, salt stress interferes with photosynthetic activity and respiration; impairs ion homeostasis and osmotic and hormonal balance; and reduces enzyme activity, protein and nucleic acid synthesis, and organic solute accumulation during plant growth and development [7]. Consequently, the enhancement of resistance to salinity in horticultural plants is currently an urgent requirement.

To survive such stress, plants have evolved different mechanisms. One of the main ways is to regulate the dynamic changes in plant gas signaling. It is reported that the signaling of gases, such as nitric oxide (NO) [8], hydrogen gas (H_2_) [9], hydrogen sulfide (H_2_S) [10], and carbon monoxide (CO) [11], play a vitally important role in responding to salt stress. In addition, plant hormones can effectively alleviate salt-stress damage, thereby promoting the adaptation to unfavorable growth conditions. For example, brassinolide (BL) is a natural product. The exogenous application of BL donor brassinosteroids (BRs) can not only promote plant growth and development but also reduce the damage caused by abiotic stresses such as salt [12] and drought [13]. Abscisic acid (ABA) is an important plant hormone with a critical role in the regulation of salt stress responses [14]. Exposure to salt stress induces the accumulation of ABA, resulting in increased tolerance [15]. This is also reported in auxin, cytokinins (CK), jasmonate (JA), gibberellin (GA), and ethylene (ET) for the alleviation of salt stress in plants [16]. Strigolactones (SLs), a class of carotenoid-derived phytohormones, have recently been identified as a new class of phytohormones. Mostofa et al. [13] have reported that SLs regulate plant adaptation to abiotic stresses, particularly nutrient deficiency, drought, and salinity. Meanwhile, SLs regulate various physiological and molecular processes during the adaptation of plants to abiotic stresses, such as root development, shoot branching, reproductive development, and leaf senescence [13].

Seed germination is an important biological process and a considerable stage in agricultural production, which is crucial for plant life cycles and crop production. Seed quality and seed germination evenness directly affect the final productivity. Thus, the regulation mechanism of the seed germination process is one of the important fields of biological research. This critical developmental step is regulated by diverse endogenous and exogenous factors [17]. Previous results reveal that seed germination is positively regulated by some novel singling molecules, such as H_2_S [18], H_2_O_2_ [19], Ga^2+^ [19], ABA [19], NO [20], and CO [21]. Additionally, an increasing number of studies have recently reported that plant hormones promote seed germination, such as ABA, auxin, ET, GA, CK, JA, and BRs [22]. Simultaneously, singling molecules [22] or plant hormones [23] alleviate the inhibition of seed germination caused by a series of abiotic stresses. SLs promote Arabidopsis seed germination under high temperature stress [24]. Tomato seedlings are resistant to the adverse effects of high salt stress when SLs, a synthetic strigolactone (GR24), are applied [25]. Limited data are available on the nature of strigolactone (SL) actions in seed germination under salt stress. In the present study, we investigate the impact of treatments with GR24 on cucumber seed germination under salt stress. In addition to the impact, we also evaluate the regulatory mechanisms of GR24 during cucumber seed germination under salt stress from the aspect of the antioxidant system. 

## 2. Materials and Methods

### 2.1. Plant Material and Treatment

Seeds of cucumber (*Cucumis sativus* L. ‘Xinchun NO. 4’) are selected based on uniformity in size and health. Cucumber seeds are soaked in 0.1% sodium hypochlorite solution for 2 min and then placed in sterile Petri plates on filter paper moistened with distilled water (the control) and different treatments for 48 h: firstly, NaCl (1, 10, 50, and 100 mM); secondly, NaCl (50 mM) + GR24 (SLs synthetic analogs; 1, 5, 10, and 20 μM); thirdly, distilled water (the control), NaCl (50 mM), NaCl (50 mM) + GR24 (5 μM), and NaCl (50 mM) + TIS108 (an inhibitor of strigolactone synthesis; 1 μM). They are allowed to germinate in the dark in an illumination incubator at 29 ± 1 °C for 48 h. Each process is set to three replicates. After 48 h, the seeds are freshly preserved at −80 °C refrigerator and used for the following analysis.

### 2.2. Determination of Germination Rate, Radicle Long, and Lateral Root Number of Seeds

When the germ length exceeds half of the length of seeds, it is defined as germination according to the study of Huang et al. 2021 [26]. The germination rate is calculated, the radicle long is measured from the root tip to the germ and hypocotyl junction, and lateral root number is recorded. Seed vitality is determined by triphenyl tetrazolium chloride (TTC) solution [27]. The cut seeds are immersed under 0.5% (*w*/*v*) TTC solution in a Petri dish and incubated at 29 ± 1 °C for 2 h. Then, the TTC solution is removed, and the seeds are washed with distilled water three times and photographed. Thirty seeds are analyzed for each treatment with three replicates.

### 2.3. Determination of MDA, H_2_O_2_, O_2_^−^, and Proline Contents

The malondialdehyde (MDA) content is analyzed by using the method described by Ma et al. [28], with several modifications. Germinated seeds (0.5 g) are homogenized in 5 mL of 10% trichloroacetic acid (TCA) and then centrifuged at 10,000× *g* for 10 min at 4 °C. The 1 mL supernatant liquor taken is added to 1 mL of 0.6% 2-thiobarbituric acid (TBA). The mixture is boiled in a water bath at 100 °C for 20 min and then centrifuged for 15 min at 10,000× *g*. The absorbance of the supernatant is measured at 450, 532, and 600 nm after cooling to room temperature. MDA content (10^−3^ μmol·g^−1^) = 6.45 × (OD_532_ − OD_600_) − 0.56 × OD_450_. 

The H_2_O_2_ content is determined as described by Prochazkova et al. [29], with slight modifications. Germinated seeds (0.5 g) are homogenized in cold 100% acetone (5 mL) and then centrifuged at 12,000× *g* for 20 min at 4 °C. The supernatant (1.0 mL) taken is added to a mixture consisting of 0.1 mL of 10% titanium tetrachloride and 0.2 mL of an ammonia–water mixture. The mixtures are centrifuged at 12,000× *g* for 15 min at 4 °C after a reaction for 5 min and the supernatant is discarded. H_2_SO_4_ (2 M, 3 mL) is added to dissolve the sediment; the absorbance is measured at 412 nm.

The H_2_O_2_ content is expressed as millimoles per gram of fresh weight (FW).

The method described by Hu et al. [30] is adopted to determine O_2_^−^ content. Samples (0.5 g) are homogenized with 5 mL of extraction buffer that contained 1 mM ethylene-diaminetetraacetic acid (EDTA), 0.3% Triton X-100, and 2% polyvinylpyrrolidone (PVP). Subsequently, the mixtures are centrifuged at 12,000× *g* for 20 min at 4 °C. The mixture comprises 1 mL of 50 mM potassium phosphate buffer, 1 mL of 1 mM hydroxylamine hydrochloride, and 1 mL of crude extract in a reaction tube and incubates at 25 °C for 1 h. After, the mixture is continually incubated at 25 °C for 20 min when 1 mL of 17 mM sulfanilic acid and 1 mL of 7 mM α -naphthylamine are added to the reaction tube. Lastly, the absorbance is measured at 530 nm and the micromoles per minute per gram of FW are used to express the O_2_^−^ content.

Proline content is measured by the Storey and Jones method with some modifications [31]. The 5 mL 3% sulfosalicylic acid is added to 0.5 g germinated seeds, which are used to obtain the proline extract by boiling water bath for 15 min. The glacial acetic acid and acid ninhydrin (2 mL) is added to the proline extract, which is heated in a boiling water bath for 30 min. Then, the extraction solution is cooled quickly on ice to room temperature and toluene (5 mL) is added. Finally, the absorbance is measured at 520 nm. Proline content is expressed as milligram per gram of FW.

### 2.4. Determination of AsA and GSH Contents

The titrimetric method with 2,6-dicloro-phenol-indophenol reagent described by Contreras-Calderón et al. [32], with several modifications, is used. The germinated seeds (0.5 g) and 5 mL of 2% oxalic acid solution are mixed together. The mixture is homogenized, diluted to 50 mL with 2% oxalic acid solution, and then filtered. The filtered solution (10 mL) is titrated with 0.01% 2,6-dichloro-phenol-indophenol solution. The final point occurs when the solution has a pink color for 30 s. The ascorbic acid content is expressed as milligrams per g of FW.

The glutathione (GSH) content is measured with an enzyme-linked immunosorbent assay (ELISA) kit (Solarbio Science & Technology, Beijing, China) according to the manufacturer’s instructions. 

### 2.5. Determination of SOD, POD, CAT, and APX Activities 

The superoxide dismutase (SOD), peroxidase (POD), catalase (CAT), and ascorbate peroxidase (APX) activities are measured with an ELISA kit (Solarbio Science & Technology, Beijing, China) according to the manufacturer’s instructions. 

### 2.6. Total RNA Extraction and Gene Expression Analysis

Total RNA in germinated seeds is extracted using TRIzol reagent described by Zhao et al. [33], with slight modifications. In brief, the samples (0.1 g) are ground into powder with a grinding miller. TRIzol (1 mL) is added to the powder and incubated for 10 min on ice. Then, 200 µL chloroform is added and incubated for 5 min; following that, the mixture is centrifuged at 12,000× *g* for 15 min at 4 °C and the supernatant is collected. An equal volume of isopropanol is mixed with supernatant, which is incubated at −20 °C for more than 1 h. Then, the mixture is centrifuged at 12,000× *g* for 15 min at 4 °C and sediment is collected. The collected sediment is washed with 75% ethanol twice. The sediment is dissolved with RNase-free ddH_2_O, which is defined as RNA. RNA quality and quantity are assessed using a NanoDrop spectrophotometer and an Agilent 2100 spectrophotometer, and more than 1 µg RNA samples are used for the cDNA synthesis. cDNA synthesis is applied with the 5 × *Evo M-MLV*RT Master Mix (AG, Changsha, China) according to the manufacturer’s instructions. The cDNA is amplified with 2 × SYBR Green Pro Taq HS Premix (AG, China). Then, gene-specific primers are shown in Table 1. The reactions are controlled by the following conditions: 30 s at 95 °C, then 5 s at 95 °C, and 30 s at 60 °C for 40 cycles. The expression levels of *SOD*, *POD*, *CAT*, *APX*, and *GRX2* genes are presented as values relative to the corresponding control samples under the indicated conditions, with normalization of data to the geometric average of internal tomato *Actin* [34]. The expression level of the gene is calculated by 2^−∆∆CT^. Briefly, ∆CT = CT (target gene) − CT (internal reference gene). ∆∆CT = ∆CT (test group) − ∆CT (control group). Each sample is set to three biological replicates.

### 2.7. Statistical Analysis

Where indicated, all the experiments are performed with three replicates and results are expressed as mean ± SE. All data are analyzed using Microsoft Excel (Redmond, WA, USA). Using SPSS 22.0 (SPSS Institute Inc., Chicago, IL, USA), Duncan’s multiple range is used to compare mean values at the *p* < 0.05 significance level. All figures are prepared with OriginPro 2017 (OriginLab Institute Inc., Northampton, MA, USA).

## 3. Results

### 3.1. Salt Stress Inhibits Seed Germination in Cucumber

**Figure 1:**
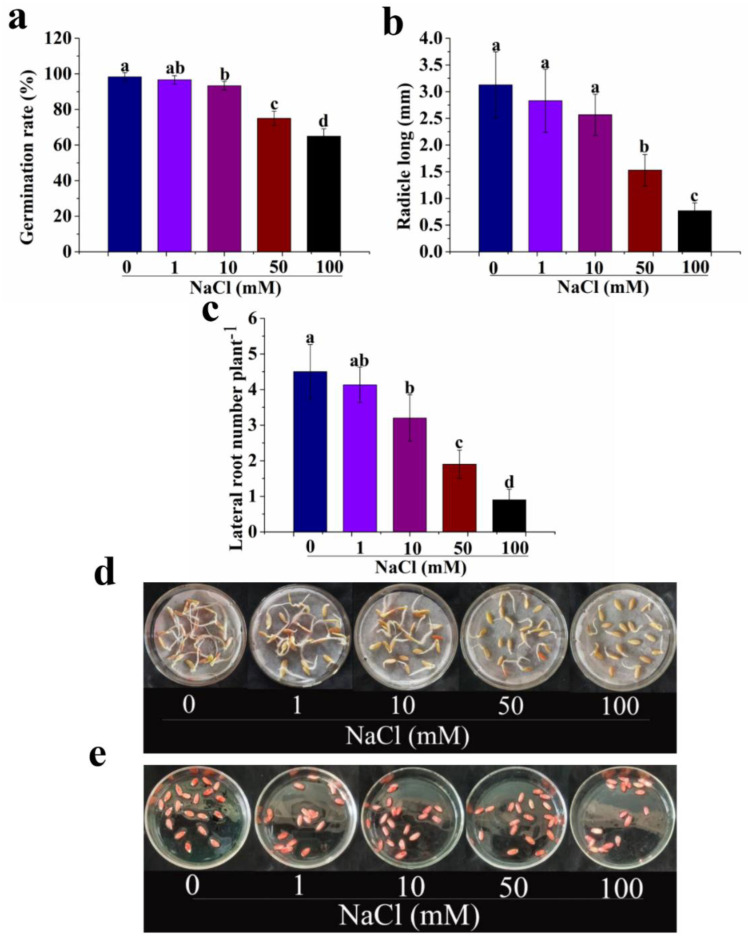
Effects of different NaCl concentrations (0, 1, 10, 50, and 100 mM) on germination rate (**a**), radicle long (**b**), lateral root number (**c**), and seed vitality (**d**,**e**), respectively. The values (means ± SE) are the averages of three independent experiments (n = 20). Bars not sharing the same letters indicate statistically significant differences by Duncan’s multiple range test (*p* < 0.05).

As shown in Figure 1a, the germination rate of seeds significantly decreases with the increase in NaCl concentrations. Compared with the control (0 mM NaCl), the germination rate of seeds treated with 1, 10, 50, or 100 mM NaCl decreases by 1.68, 5.08, 23.73, and 33.90%, respectively. Likewise, the application of NaCl of different concentrations from 1 to 100 mM decreases the radicle long and lateral root number of seeds (Figure 1a,b). The germination rate, radicle long, and lateral root number of seeds treated with 50 mM NaCl all exhibit a significant decrease and increase in comparison with 10 mM NaCl and 100 mM NaCl, respectively (Figure 1a–c). These results are in full agreement with the changes of seeds revealed in Figure 1d and seed vitality in Figure 1e. Therefore, treatments with 1–10 mM, 50 mM, and 100 mM NaCl could be termed as mild, moderate, and severe salt stress, respectively. As 50 mM NaCl induces moderate salt stress, the concentration is used for the next experiment.

### 3.2. Effects of Exogenous GR24 on Seed Germination under Salt Stress

**Figure 2:**
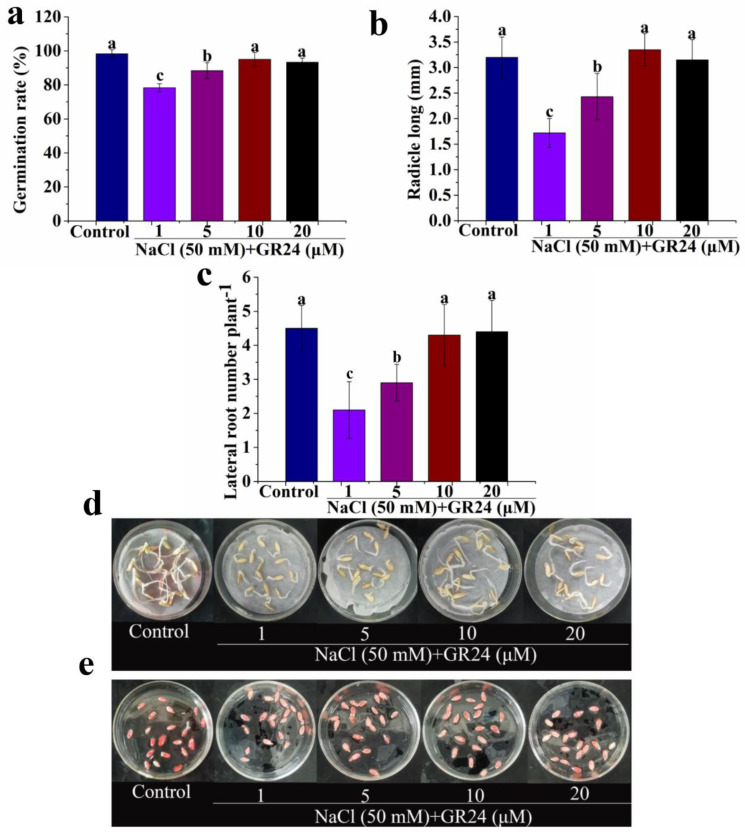
Effects of different concentrations of GR24 on germination rate (**a**), radicle long (**b**), lateral root number (**c**), and seed vitality (**d**,**e**) under salt stress, respectively. The values (means ± SE) are the averages of three independent experiments (n = 20). Bars not sharing the same letters indicate statistically significant differences by Duncan’s multiple range test (*p* < 0.05).

To understand the effects of exogenous GR24 on seed germination under salt stress, we performed dose-response experiments with GR24. As shown in Figure 1, the germination of cucumber seeds is significantly decreased under salt stress in comparison with the control. However, different concentrations of GR24 (1–20 μM) significantly alleviates the decrease in germination rate, radicle long, lateral root number, and seed vitality of seeds caused by salt stress (Figure 2). Moreover, treatments with 10 and 20 μM GR24 result in the increase almost reaching the level of the control (Figure 2). These results show that the effect of GR24 on seed germination under salt condition was dose-dependent (Figure 2). Therefore, GR24 (10 μM), being the most effective under our experimental conditions, is used further for studies. 

**Figure 3:**
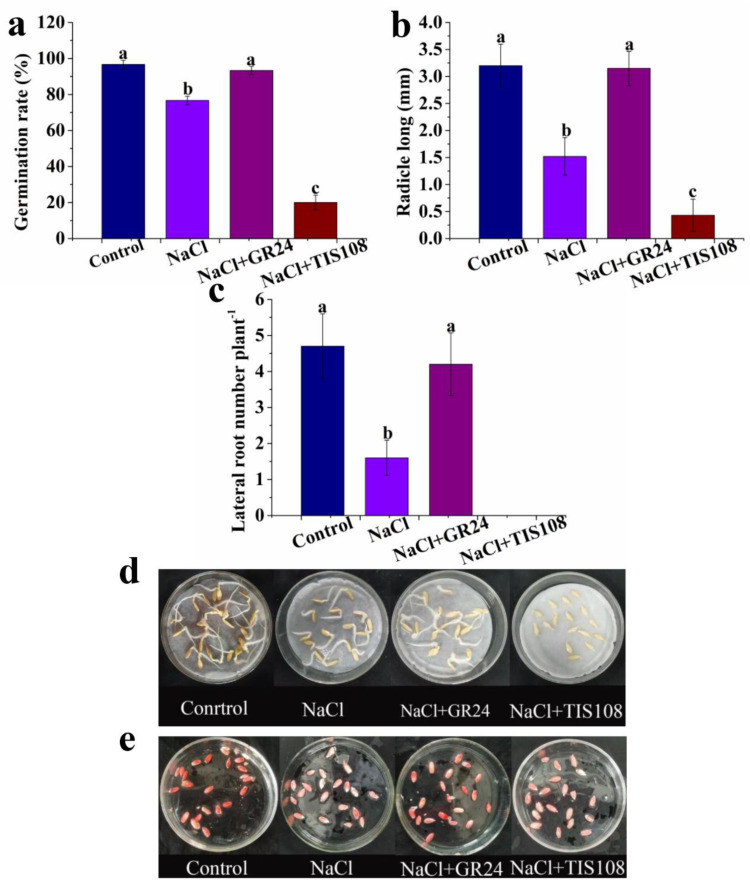
Effects of GR24 and TIS108 on germination rate (**a**), radicle long (**b**), lateral root number (**c**), and seed vitality (**d**,**e**) under salt stress, respectively. The values (means ± SE) are the averages of three independent experiments (n = 20). Bars not sharing the same letters indicate statistically significant differences by Duncan’s multiple range test (*p* < 0.05).

To further testify to the alleviation of GR24 in seed germination under salt stress, TIS108 (an inhibitor of strigolactone synthesis) is applied. The decreases in the germination rate, radicle long, lateral root number, and vitality of seeds caused by NaCl were distinctly increased by NaCl + GR24 (Figure 3). However, when TIS108 is added to the NaCl treatment, the germination rate, radicle long, lateral root number, and vitality of seeds are significantly decreased (Figure 3). These results demonstrate that GR24 can improve seed germination in cucumber under salt stress.

### 3.3. Effects of GR24 on MDA, H_2_O_2_, O_2_^−^, and Proline Contents during Seed Germination under Salt Stress

**Figure 4:**
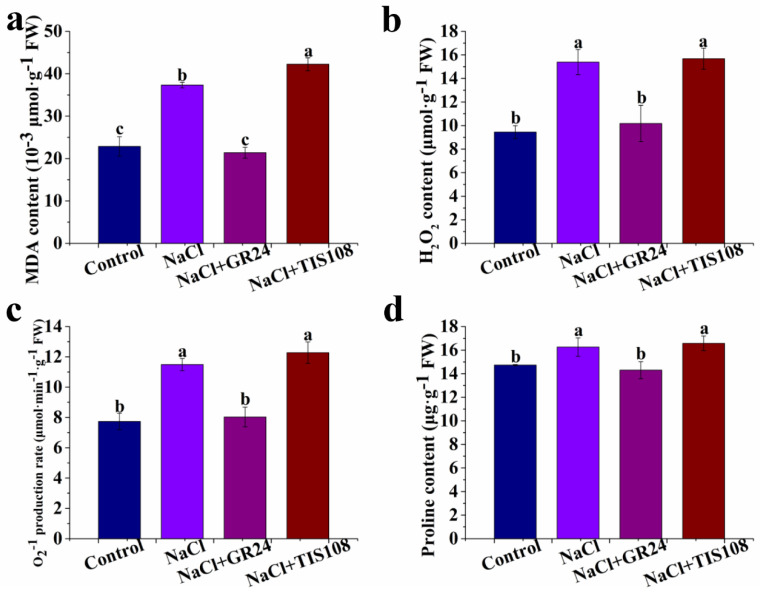
Effects of GR24 and TIS108 on MDA (**a**), H_2_O_2_ (**b**), O_2_^−^ (**c**), and proline (**d**) contents of cucumber seeds under salt stress, respectively. The values (means ± SE) are the averages of three independent experiments (n = 20). Bars not sharing the same letters indicate statistically significant differences by Duncan’s multiple range test (*p* < 0.05). MDA, Malondialdehyde; H_2_O_2_, hydrogen peroxide; O_2_^−^, superoxide anion.

Compared with the control (distilled water), the treatment of NaCl results in an obvious enhancement in the MDA, H_2_O_2_, O_2_^−^, and proline contents (Figure 4). The MDA, H_2_O_2_, O_2_^−^, and proline contents of the treatment with NaCl plus GR24 is lower than that of the NaCl treatment (Figure 4). Treatment with NaCl + TIS108 causes a significant increase in MDA content compared with treatment with NaCl (Figure 4a). NaCl + TIS108 treatment has no significant influence on the H_2_O_2_, O_2_^−^, and proline contents under salt stress (Figure 4). These results indicate that the MDA, H_2_O_2_, O_2_^−^, and proline contents of the seeds are decreased by GR24 under salt stress, suggesting that GR24 improves salt-inhibited seed germination by inhibiting lipid peroxidation.

### 3.4. Effects of GR24 on AsA and GSH Contents during Seed Germination under Salt Stress

**Figure 5:**
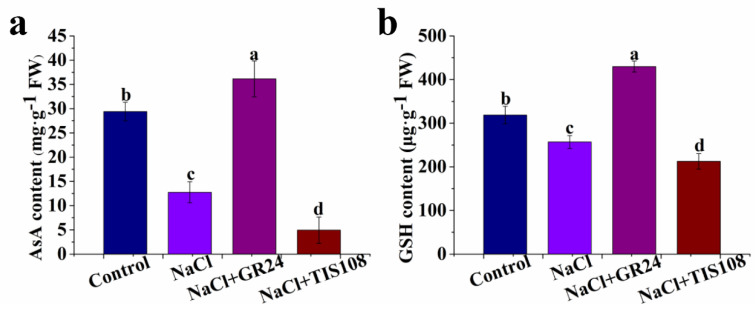
Effects of GR24 and TIS108 on AsA (**a**) and GSH (**b**) contents of cucumber seeds under salt stress, respectively. The values (means ± SE) are the averages of three independent experiments (n = 20). Bars not sharing the same letters indicate statistically significant differences by Duncan’s multiple range test (*p* < 0.05). AsA, ascorbate; GSH, glutathione.

During seed germination, the ascorbic acid (AsA) content is declined by salt stress. Compared with the NaCl treatment, the content of AsA in the treatment with NaCl + GR24 increases by 184% (Figure 5a). However, the AsA content of the NaCl + GR24 treatment is more than that of the NaCl + TIS108 treatment (Figure 5a). The change in the GSH content is similar to that in the AsA content (Figure 5). Under salt stress, the increase in the GSH content decreases significantly when TIS108 is added (Figure 5b). The increase in the GSH content caused by NaCl + GR24 is distinctly decreased by NaCl + TIS108 (Figure 5b). These results demonstrate that endogenous GR24 increases the antioxidant AsA and GSH contents under salt condition.

### 3.5. Effects of GR24 on SOD, POD, CAT, and APX Activities during Seed Germination under Salt Stress

**Figure 6:**
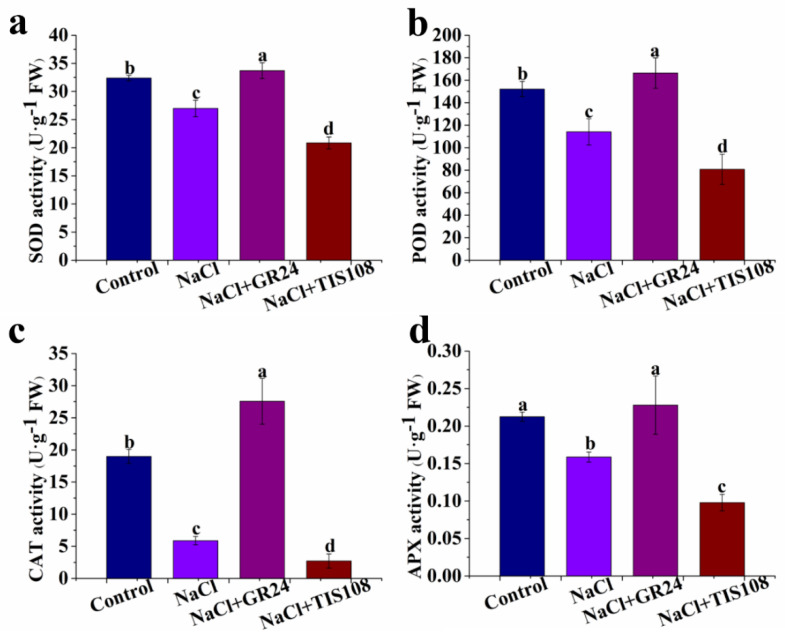
Effects of GR24 and TIS108 on SOD (**a**), POD (**b**), CAT (**c**), and APX (**d**) activities of cucumber seeds under salt stress, respectively. The values (means ± SE) are the averages of three independent experiments (n = 20). Bars not sharing the same letters indicate statistically significant differences by Duncan’s multiple range test (*p* < 0.05). SOD, superoxide dismutase; POD, peroxidase; CAT, catalase; APX, ascorbate peroxidase.

Compared with the control, the NaCl treatment induces lower activities of SOD, POD, CAT, and APX. When GR24 is added to NaCl, SOD, POD, CAT, and APX activities remarkably increase (Figure 6). However, a significant decrease in SOD, POD, CAT, and APX activities in the NaCl + TIS108 treatment is observed (Figure 6). These results suggest that GR24 alleviates seed germination under salt stress by increasing the antioxidant enzymes’, SOD, POD, CAT, and APX, activities. 

### 3.6. Effects of GR24 on the Gene Expression Level Related to Antioxidant Enzymes during Seed Germination under Salt Stress

**Figure 7:**
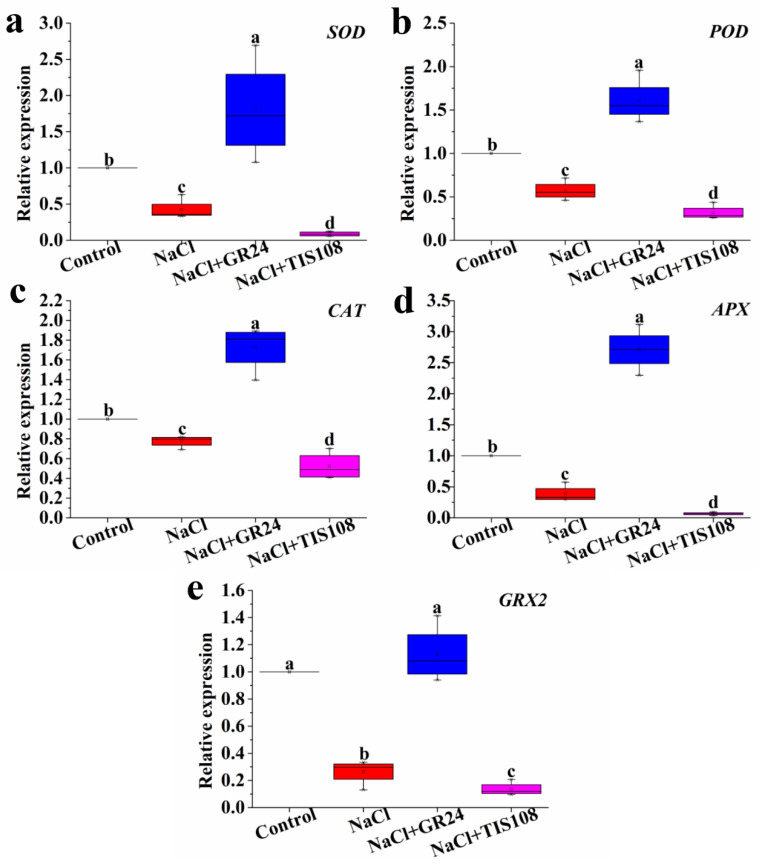
Effects of GR24 and TIS108 on the expression levels of *SOD* (**a**), *POD* (**b**), *CAT* (**c**), *APX* (**d**), and *GRX2* (**e**) genes of cucumber seeds under salt stress, respectively. The values (means ± SE) are the averages of three independent experiments (n = 20). Bars not sharing the same letters indicate statistically significant differences by Duncan’s multiple range test (*p* < 0.05).

As shown in Figure 7, the expression levels of *SOD*, *POD*, *CAT*, *APX*, and *GRX2* genes are significantly down-regulated under salt stress. The NaCl + GR24 treatment shows higher *SOD*, *POD*, *CAT*, *APX*, and *GRX2* expression than the NaCl treatment (Figure 7). Conversely, the expression levels of *SOD*, *POD*, *CAT*, *APX*, and *GRX2* genes are decreased by the NaCl + TIS108 treatment (Figure 7). These results demonstrate that the genes related to antioxidant enzymes are involved in the alleviation of salt-induced seed germination.

## 4. Discussion

Seed germination is one of the stages most sensitive to adverse environmental conditions; meanwhile, it is also an important biological process that determines plant life cycles and crop production [35]. Salt stress is an essential limiting element in seed germination, which leads to a reduction in germination rates and a delay in the initiation of germination [36,37,38]. The germination rate, radicle long, lateral root number, and vitality of seeds are commonly used as indicators that reflect seed germination and vigor [39]. In this study, the germination rate, radicle long, and lateral root number, as well as seed vitality, are significantly decreased by an NaCl treatment (Figure 1), which further confirms the negative effect of salt stress on seed germination. Previous studies indicate that SLs improve seed germination in plants [40]. This study examines whether SLs positively influence cucumber seed germination under salt stress. In the present study, GR24 responds to salt stress in a dose-dependent manner during seed germination, and the greatest biological response is observed in 10 µM GR24 treatment (Figure 2). However, the SLs’ synthesis inhibitor TIS108 reserves the positive roles of GR24 in seed germination under salt stress (Figure 3). These results imply that SLs alleviate the inhibition of cucumber seed germination caused by salt stress. Genes in the Suppressor of MAX2 1-Like (SMXL) family are known downstream elements of the SLs’ signaling pathways; they regulate the salt stress responses of soybean during seed germination, potentially via MAX2-mediated SLs-dependent and -independent pathways [41]. The application of GR24 also enhances salt resistance in rice seedlings [42]. The exogenous application of GR24 enhances plant tolerance to low light stress, thus improving tomato seedlings growth [43]. The applied exogenous GR24 could effectively alleviate salinity–alkalinity stress in apple seedlings [44]. Exogenous GR24 also alleviates salt stress in cucumber seedlings [45]. Exogenous GR24 might alleviate the low light stress-induced growth inhibition of cucumber seedlings [46]. Based on the results mentioned above, SLs may be applied as a potential approach to reduce the negative impact of abiotic stress on seed germination and seedling growth in plants.

Salt toxicity can cause an increase in the formation and accumulation of ROS, such as H_2_O_2_ and O_2_^−^, which directly or indirectly damages the cellular components, enzymes, and biological membranes, and even cell death [38,47,48,49]. MDA is formed by the reaction of ROS with lipid molecules of tissues and is toxic to biomacromolecules. It is well known that MDA, H_2_O_2_, and O_2_^−^ are often used as indicators for oxidative damage in plants under various environmental conditions [35,50,51]. Additionally, proline as an organic solute is well known for its osmotic adjustment activity and role in enhancing salt tolerance through the protection of cellular membranes and enzyme integrity [52]. Our results showed that NaCl stress-induced oxidative stress in cucumber is indicated by the increase in MDA, H_2_O_2_, O_2_^−^, and proline levels during seed germination (Figure 4). These findings are in line with the salinity-induced oxidative stress described in previous studies in rice [53], maize [54], cucumber [55], bean [47], and tomato [56]. However, GR24 treatment decreases the MDA, H_2_O_2_, O_2_^−^, and proline contents of seeds under salt stress (Figure 4). When GR24 is inhibited, the increased MDA, H_2_O_2_, O_2_^−^, and proline contents are decreased, indicating that SLs can alleviate oxidative damage caused by salt stress under seed germination by decreasing MDA, H_2_O_2_, O_2_^−^, and proline levels. *MORE AXILLARY GROWTH2* (*MAX2*) is a key gene in the signal transduction pathway of SLs. Zhang et al. [57] proved that *CsMAX2*-overexpressing lines had a low MDA content after salt, drought, and ABA stress, which is lower than that of WT plants. The proline content and lipid peroxidation of seeds primed with GR24 is enhanced or reduced, respectively, under heat stress [58]. Under low light stress, GR24 also increases the accumulation of carbohydrates and reduces the levels of H_2_O_2_ and MDA in cucumber seedlings [46]. Exogenous GR24 pretreatment can scavenge salt-induced excessive ROS, thus alleviating oxidative stress under salt stress in cucumber [45]. This also further suggests that H_2_O_2_ pathways might play a positive role in exogenous GR24 alleviating salt stress in cucumber seedlings [45]. GR24 application reduces MDA and H_2_O_2_ contents in low light-stressed tomato seedlings [43]. It is speculated that the SLs might be directly involved in the regulation of stress resistance by reducing MDA, H_2_O_2_, O_2_^−^, and proline contents, thus alleviating oxidative stress in plants.

To protect themselves against oxidative stress, plants have evolved ROS scavenging systems [59]. It is well known that the coordinated action of antioxidant substances, including AsA and GSH and antioxidant enzymes such as SOD, CAT, APX and POD, plays a significant role in scavenging ROS to protect cell membranes, which is thought to be a major mechanism of resistance to salt stress in plants [38,47,49]. Our results demonstrate a significant decrease in the AsA and GSH contents in NaCl-treated plants compared with non-treated plants (Figure 5). Cucumber seeds treated with GR24+NaCl have higher AsA and GSH contents than those in NaCl treatment alone, while the increase is decreased by TIS108 (Figure 5). The results suggest that SLs increase the contents of the non-enzyme substances, AsA and GSH, to alleviate salt stress-caused oxidative damage during seed germination. GR24 treatment effectively increases AsA levels in tomato seedlings under salt stress, while TIS108 blocks the increase in AsA content [25]. Thus, SLs may play an important role in improving salinity tolerance in plants by enhancing antioxidant capacity. GR24 also enhances the activities of SOD, APX, and POD antioxidant enzymes in lupine seedlings under heat stress [58]. Our results also show that GR24 treatment with a suitable concentration significantly improves the activities of SOD, CAT, APX, and POD in cucumber seeds under salt stress (Figure 6). The increased enzyme activities match the lower MDA, H_2_O_2_, and O_2_^−^ levels (Figure 3 and Figure 6). Additionally, the expression levels of the *SOD*, *POD*, *CAT*, *APX*, and *GRX2* genes are up-regulated by GR24, but their expression is down-regulated by TIS108 under salt stress (Figure 7). Under salinity–alkalinity stress, exogenous GR24 increases the activities of SOD, POD, and CAT enzymes, thereby eliminating ROS production and improving the salinity–alkalinity tolerance of apple [44]. RNA-seq analysis revealed that differentially expressed genes related to oxidation and the antioxidant system are up-regulated by exogenous application GR24 under salt stress in cucumber seedlings [45]. These results give strong evidence that a higher antioxidant defense capacity is one of the important mechanisms for exogenous GR24 to alleviate oxidative stress and improve stress tolerance.

## 5. Conclusions

In conclusion, this study clarifies the vital role of SLs in alleviating salt stress during cucumber seed germination. Moreover, the results reveal a mechanism of SLs-relieved salt toxicity during seed germination, which involves an antioxidant system: GR24 decreases the increase in MDA, H_2_O_2_, O_2_^−^, and proline contents caused by salt stress by increasing AsA and GSH contents and enhancing SOD, POD, POD, and APX activities, following the up-regulation of *POD*, *CAT*, *APX*, and *GRX2* genes during seed germination. Collectively, these findings provide new insights into the biological functions of SLs in plant growth and stress response. However, the mechanisms underlying SLs and abiotic stress are quite complex, and future works should further explore the deeper mechanisms of SLs-alleviated seed germination under salt stress conditions, including the mining of critical genes and receptors.

## Figures and Tables

**Table 1:** Primer sequence for qRT-PCR.

Gene Name	Gene ID	Primer Sequence (5′–3′)
*SOD*	LOC101215417	ACGGGTAATGTTTCTGGTCTCAAGC CATGCAGCCATTGGTTGTGTCAC
*POD*	LOC101212957	AGACGGTGGTGGGAGTGGAATC GAGCAACGATGTCAGCACAAGAAAC
*CAT*	LOC101216662	GAGCAACGATGTCAGCACAAGAAAC TCCAAGACGGTGCCTCTGAGTATC
*APX*	LOC101212957	AAAGTGCTACCCTGTTGTGAGTGAG AAAGTGCTACCCTGTTGTGAGTGAG
*GRX2*	LOC101205064	TCGGTGGTTGCGATGGTAAGAAC GCTGACCTCTGTGATGCTTCTCTG
*Actin*	LOC101220617	F: TTCTGGTGATGG TGTGAGTC R: GGCAGTGGTGGTGAACATG

## Data Availability

Data is contained within the article.
